# Clinical Characteristics and Potential Mechanisms in Patients with Abnormal Liver Function Indices and Elevated Serum IgG4

**DOI:** 10.1155/2022/7194826

**Published:** 2022-08-25

**Authors:** Jing Wang, Yue Zhang, Dandan Jiang, Lu Zhou, Bangmao Wang

**Affiliations:** ^1^Department of Gastroenterology and Hepatology, Tianjin Medical University General Hospital, Tianjin 300052, China; ^2^Inner Mongolia Institute of Digestive Diseases, The Second Affiliated Hospital of Baotou Medical College, Baotou 014030, China

## Abstract

**Objective:**

We analyzed the etiological classification and clinical characteristics of patients with abnormal liver function indices and elevated serum IgG4 levels and investigated the effects of intrahepatic follicular helper T cell (Tfh) infiltration and serum IL-21.

**Methods:**

Clinical data (age, sex, past history, clinical manifestations, laboratory tests, imaging, diagnosis, and treatment) and etiology of liver injury from 136 patients were analyzed. We compared the general condition, clinical characteristics, and laboratory tests of 19 AIH (autoimmune hepatitis) patients with elevated serum IgG4 levels with those of 20 AIH patients with normal serum IgG4 levels admitted at the same time. Five patients with AIH and elevated serum IgG4 levels and five AIH patients with normal IgG4 levels were matched by sex, age, and liver function, and Tfh infiltration in liver biopsy tissues of patients in both groups was determined by immunofluorescence staining. Five AIH patients with elevated serum IgG4 levels were selected for measurement of serum interleukin-21 (IL-21) levels by enzyme-linked immunosorbent assay (ELISA), seventeen AIH patients with normal serum IgG4 were matched by sex, age, and liver function indices, and 29 physically healthy individuals matched by sex and age were selected as the control group. The changes in patients with IgG4-RD and abnormal liver function before and after glucocorticoid treatment were measured.

**Results:**

Patients (136) with abnormal liver function indices and elevated serum IgG4 levels were diagnosed with liver disease of different etiologies. IgG4-related disease was the most frequent, followed by AIH and malignancy. Abnormal liver function indices with high serum IgG4 were most commonly seen as elevated gamma glutamyl transferase (GGT). The AIH group with elevated serum IgG4 had increased intrahepatic levels of Tfh. IL-21 in AIH patients with elevated IgG4 was higher than in patients with normal IgG4 and healthy controls. Patients (*n* = 28) with abnormal liver function indices and IgG4-related disease received glucocorticoid therapy for six months, and ALT, AST, ALKP, GGT, TBil, DBil, IgG, IgG4, and IgE were significantly lower after treatment.

**Conclusions:**

Elevated serum IgG4 was seen in patients with abnormal liver function indices with diverse causes. Tfh infiltration and increased IL-21 production may be related to the pathogenesis of AIH with elevated serum IgG4. Glucocorticoid therapy is effective in patients with abnormal liver function indices and IgG4-related disease. Assessing immune function in patients with abnormal liver function indices and elevated serum IgG4 levels should facilitate diagnosis and treatment of the disease.

## 1. Introduction

IgG4-related disease (IgG4-RD) is characterized by immune-mediated inflammation with fibrosis. The hallmarks of IgG4-RD are the presence of marked lymphoplasmacytic infiltration of affected tissues and organs, predominantly by IgG4-positive plasma cells, and elevated serum IgG4 levels accompanied by matricial fibrosis and occlusive phlebitis. Most patients improve significantly after glucocorticoid treatment but are prone to recurrence. IgG4-RD occurs primarily in middle-aged and elderly men, but the etiology and the immune mechanism are still unclear. The organs known to be involved include the pancreas, bile ducts, lacrimal or salivary glands, retroperitoneal tissue, central nervous system, thyroid, lungs, and liver. To date, seven types of liver injury have been reported in IgG4-RD: (1) IgG4-related inflammatory pseudo-tumor of the liver [1]; (2) IgG4-associated autoimmune hepatitis (IgG4-AIH) [2]; (3) type 1 autoimmune pancreatitis (AIP) with liver injury [3]; (4) IgG4-related sclerosing cholangitis (IgG4-SC) [4]; (5) primary biliary cholangitis (PBC) with IgG4^+^ cell infiltration in hepatic nodular lymphadenopathy [5]; (6) IgG4-RD with increased numbers of eosinophils in the blood causing liver damage [6]; and (7) IgG4-RD with other organs (except digestive) as the primary organ. Among these conditions, type 1 AIP liver injury has been the most intensively studied. A number of pathological changes are commonly seen in type 1 AIP: periportal sclerosis, bile duct injury, inflammation of the portal area, lobular hepatitis, and cholestasis [7]. However, the disease spectrum in patients with non-IgG4-RD, who also have elevated serum IgG4 levels and liver injury, remains to be established. Furthermore, the immune response in the liver with increased serum IgG4 levels as well as the mechanism of serum IgG4 elevation is unclear.

Follicular helper *T* (Tfh) cells are an immune cell type that has received much attention in the last decade. As a subclass of *T* helper (Th) cells, Tfh cells differentiate upon stimulation by IL-21, secrete large quantities of IL-21, and promote the conversion of B cells to plasma cells, thereby elevating IgG4 [8]. In this study, we evaluated the disease spectrum of patients with liver injury and elevated serum IgG4 levels. We also discussed the etiology and diagnosis, analyzed laboratory tests and liver pathology, and measured intrahepatic infiltration of Tfh cells and circulating IL-21. This information will provide a reference for the diagnosis and treatment of patients with liver injury and elevated serum IgG4 levels.

## 2. Subjects and Methods

### 2.1. Flowchart of Patient Enrollment and Study Design

**Figure 1:**
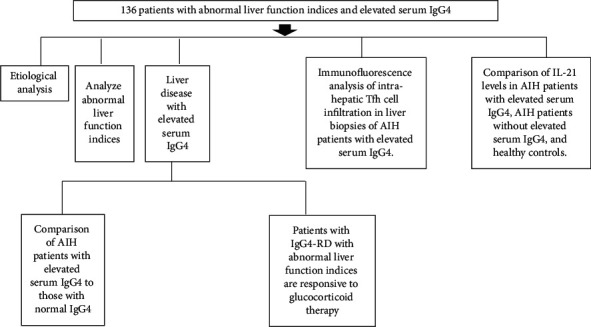
Flowchart depicting the patient enrollment and study design.

The flowchart of patient enrollment and study design is provided in Figure 1.

### 2.2. Subjects

The clinical data of 136 patients with abnormal liver function indices and elevated serum IgG4 level (>2.01 g/L) who were hospitalized at Tianjin Medical University General Hospital from May 2012 to August 2020 with complete medical records and follow-up data were analyzed retrospectively. Physically healthy individuals and autoimmune hepatitis (AIH) patients with normal serum IgG4 levels were included as controls. This study was approved by the Ethics Committee of the General Hospital of Tianjin Medical University (IRB2015-YX-002).

### 2.3. Study Indices

Clinical data (age, sex, past history, clinical manifestations, laboratory tests, imaging, diagnosis, and treatment) and etiologies of liver injury from 136 patients were obtained. We compared the general condition, clinical characteristics, and laboratory tests of 19 AIH patients with elevated serum IgG4 levels with those of 20 AIH patients with normal serum IgG4 levels admitted at the same time. Five patients with AIH and elevated serum IgG4 levels and five AIH patients with normal IgG4 levels were matched by sex, age, and liver function. Tfh infiltration in liver biopsy tissues of patients in both groups was determined by immunofluorescence staining. Five AIH patients with elevated serum IgG4 levels were selected for measurement of serum interleukin-21 (IL-21) levels by enzyme-linked immunosorbent assay (ELISA). Seventeen AIH patients with normal serum IgG4 were matched by sex, age, and liver function indices, and 29 physically healthy individuals matched by sex and age were selected as the control group. The changes in patients with IgG4-RD complicated with abnormal liver function before and after glucocorticoid treatment were measured.

### 2.4. Diagnostic Criteria

IgG4-RD was diagnosed according to the diagnostic criteria published in Japan in 2012 [9]. Due to different laboratory testing standards, the threshold level for elevated serum IgG4 in this study was >2.01 g/L. AIH was diagnosed using scoring criteria recommended by the American Association for the Study of Liver Diseases (AASLD) (2010) [10]. The overlap syndrome (OS) was diagnosed in accordance with the characteristics of overlap between AIH and primary biliary cirrhosis (PBC) recommended by the International Autoimmune Hepatitis Group (IAIHG) [11]. Drug-induced liver injury (DILI) was diagnosed according to the Chinese Society of Hepatology (CSH) guidelines [12]. Liver injury was associated with clinical symptoms such as anorexia, nausea, vomiting, fatigue and weakness, dizziness, pain in the liver area, and jaundice. Liver function was assessed by laboratory measurement of the liver function parameters, TBil, DBil, ALT, AST, GGT, and ALKP, with values outside the normal ranges indicating abnormality.

### 2.5. Statistical Analysis

Statistical analysis was performed using SPSS 22.0 and GraphPad Prism 7.0 software. For measurement data, the mean ± standard deviation (SD) was used to represent normally distributed data, while the median (quartile range), M(Q), was used to represent non-normally distributed data. The number of cases or percentages was used to describe the count data. Measurement data that conformed to the normal distribution were analyzed by *t*-test, and the Fisher exact probability method was used for comparison of the count data between groups. *P* < 0.05 was set as the threshold for statistical significance.

## 3. Results

### 3.1. Etiological Analysis of Patients with Abnormal Liver Function Indices and Elevated Serum IgG4

Patients with abnormal liver function indices and elevated serum IgG4 (*n* = 136) were screened using the medical record management system or clinical records and classified according to sex, age, medical history, laboratory tests, histopathology, and immunological examinations. The patients were also categorized according to etiology: IgG4-related diseases (52 IgG4-RD, 38.2%), autoimmune hepatitis (19 AIH, 14.0%), malignancy (17, 12.5%), nonalcoholic fatty liver disease (6, 4.4%), viral hepatitis (4, 2.9%), alcoholic liver disease (4, 2.9%), and primary biliary cholangitis (4 PBC, 2.9%). There was one case each (0.7%) of primary sclerosing cholangitis, drug-related liver injury, liver abscess, and gallstones, and 26 patients (19.1%) had hepatic impairment of unknown etiology.

Among the 52 patients with IgG4-RD and elevated serum IgG4, 34 (65.4%) were male and 24 (46.2%) were ≥65 years of age. Organ involvement included pancreas (31), bile duct (16), retroperitoneum (7), salivary gland (3), kidney (4), submandibular gland (3), lacrimal gland (2), lung (2), and other organs (3).

### 3.2. Assay of Liver Enzymes, Bilirubin, and Bile Enzymes in Patients with Abnormal Liver Function and Elevated Serum IgG4

Among the patients with abnormal liver function indices and elevated serum IgG4 (*n* = 136), patients with elevated GGT were the most common, accounting for 86.8% (118), of which 76 were elevated 1- to 5-fold (64.4%) and 24 were elevated 10-fold (20.3%) (Table 1**)**.

### 3.3. Comparison of AIH Patients with Elevated Serum IgG4 to Those with Normal IgG4

#### 3.3.1. Comparison of the General Conditions, Clinical Symptoms, and Laboratory Test Results between AIH Patients with and without Elevated Serum IgG4

Details of the general condition, clinical symptoms, and laboratory test results of 19 AIH patients with elevated serum IgG4 and 20 AIH patients with normal serum IgG4 are shown in Table 2. Compared to the patients with normal serum IgG4, significantly (*P* < 0.05) more patients in the group with elevated serum IgG4 were male, had elevated IgE, AST ≥ 10 ULN, ALT ≥ 10 ULN, decreased C4, concomitant allergic disease, and constipation.

#### 3.3.2. Immunofluorescence Detection of Intrahepatic Tfh Cell Infiltration in Liver Biopsies of AIH Patients with Elevated Serum IgG4 and AIH Patients with Normal IgG4 Levels

**Figure 2:**
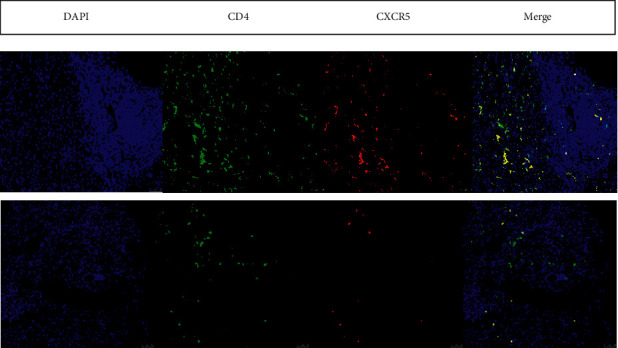
(a) Abundant intrahepatic Tfh infiltration in AIH patients with elevated serum IgG4. (b) Sparse intrahepatic Tfh infiltration in AIH patients with normal IgG4 level. Tfh localization was assessed by immunostaining and fluorescence microscopy: nuclei were stained with DAPI (blue), CD4 (green), CXCR5 (red), and the DAPI, CD4, and CXCR5 images were merged.

Five patients with AIH and elevated serum IgG4 and five AIH patients with normal IgG4 levels were matched according to sex, age, and liver function. The intrahepatic Tfh cell infiltration in liver biopsy tissue was visualized by immunofluorescence staining. The group with AIH and elevated serum IgG4 had increased infiltration of Tfh in the confluent areas compared to patients with normal IgG4 levels (Figure 2).

#### 3.3.3. Comparison of IL-21 Levels in AIH Patients with Elevated Serum IgG4, AIH Patients with Normal Serum IgG4 Levels, and Physically Healthy Controls

**Figure 3:**
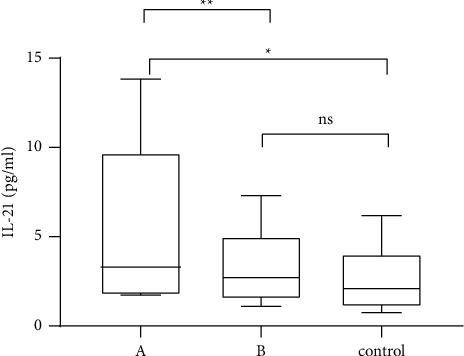
Interleukin-21 (IL-21) levels in AIH patients with elevated serum IgG4 (A), AIH patients with normal serum IgG4 (B), and healthy controls measured by ELISA.

The IL-21 levels in five AIH patients with elevated serum IgG4 (*n* = 5) were significantly higher than those in 17 AIH patients with normal IgG4 (*F* = 4.826, *P* < 0.05) and 29 sex- and age-matched healthy controls selected during the same visit period (*F* = 9.916, *P* < 0.01), with no significant difference between the latter two groups (*F* = 1.976, *P*=0.167) (Figure 3).

### 3.4. Patients with IgG4-RD Combined with Abnormal Liver Function Were Responsive to Glucocorticoid Therapy

#### 3.4.1. Changes in Liver Function Indices in Patients after Six Months of Glucocorticoid Therapy

**Figure 4:**
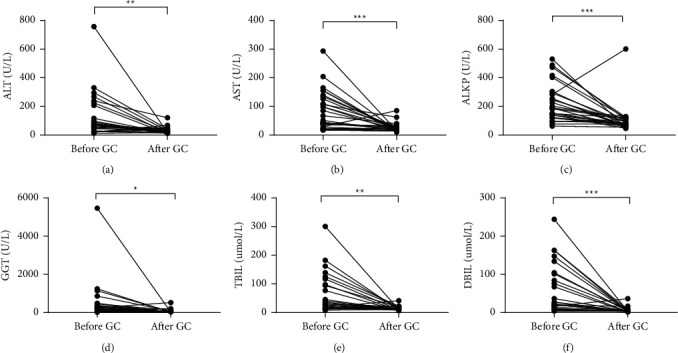
Liver function indices before and after glucocorticoid therapy in first-treated patients with IgG4-RD and abnormal liver function.

The effects of six months of glucocorticoid therapy were analyzed in 28 first-treated patients. Compared with pretreatment levels, significantly lower levels of ALT (*P*=0.0041; Figure 4(a)), AST (*P*=0.0002; Figure 4(b)), ALKP (*P*=0.0002; Figure 4(c)), GGT (*P*=0.0409; Figure 4(d)), TBil (*P*=0.0011; Figure 4(e)), and DBil (*P*=0.0003; Figure 4(f)) were detected after glucocorticoid therapy, with routine application of the hepatoprotective drugs dicyclool and magnesium isosidooxalate.

#### 3.4.2. Changes in Ig Levels in IgG4-RD Patients after Six Months of Glucocorticoid Therapy

**Figure 5:**
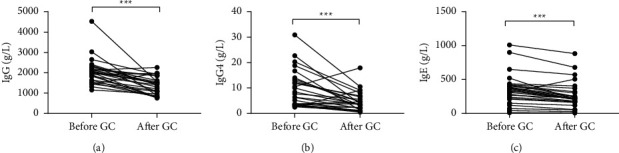
Immunoglobulin indices before and after glucocorticoid therapy in patients with IgG4-RD combined with abnormal liver function and elevated serum IgG4.

Statistical analysis of the changes of IgG, IgG4, and IgE in first-treated patients with IgG4-RD combined with abnormal liver function indices before and after glucocorticoid therapy is shown in Figure 5. The levels of IgG, IgG4, and IgE were significantly lower after glucocorticoid therapy compared with those before treatment (*P*=0.0002, *P*=0.0004, and *P*=0.0022, respectively).

## 4. Discussion

Elevated serum IgG4 levels in patients can be accompanied by varying degrees of abnormal liver function [7, 13]. The etiology of the abnormal liver function also varies. Since the mechanism is unclear, improvements in diagnosis and treatment have been slow; however, a greater clinical understanding of IgG4 regulation and IgG4-RD has resulted in a gradual increase in the number of patients identified with abnormal liver function and elevated serum IgG4 levels, including those who meet the diagnostic criteria for IgG4-RD and those who do not. Here for the first time, we studied patients with abnormal liver functions and elevated IgG4 levels and found that they manifested the following disease categories: IgG4-RD with abnormal liver functions, AIH, OS, malignancy, viral hepatitis, alcoholic liver disease, drug-related liver injury, PBC, nonalcoholic fatty liver, liver abscesses, gallstones, and liver injury of unknown etiology. It has been reported that elevated serum IgG4 can occur in most common liver diseases. However, the characteristics and immune responses of these diseases require further clarification. In this study, elevated GGT levels were common in patients with abnormal liver functions, indicating the susceptibility to synchronous bile duct damage.

Analysis of the clinical data of AIH patients with elevated serum IgG4 levels and AIH patients with normal serum IgG4 levels revealed significant differences. A comparison of 19 AIH patients with elevated serum IgG4 with 20 cases of AIH with normal serum IgG4 revealed that male patients and those with elevated IgE, AST ≥ 10 ULN, ALT ≥ 10 ULN, reduced C4, allergic disease, or constipation were more commonly seen in the AIH group with elevated serum IgG4. Therefore, we suggest that allergy, constipation, C4, and IgE may be associated with the development and progression of the diseases. We further infer that different immune mechanisms may underlie the condition of AIH patients with elevated serum IgG4 levels compared to those without elevated serum IgG4. Therefore, combined IgG4-associated AIH should be considered for male AIH patients presenting clinically with elevated ALT and AST levels.

Comparing the changes in liver function before and after the use of glucocorticoids in patients with IgG4-RD and abnormal liver function indices revealed that glucocorticoid therapy was effective, which is consistent with previous reports [14]. To further investigate the mechanism of AIH with elevated serum IgG4, we used immunofluorescence to determine intrahepatic Tfh cell infiltration in liver biopsy tissues. Tfh infiltration in liver tissue of AIH patients with elevated serum IgG4 was higher than that in AIH patients with normal serum IgG4. Furthermore, AIH patients with elevated serum IgG4 had higher IL-21 levels than those with normal serum IgG4 and healthy controls. IL-21 is essential for Tfh cell development and plays a key role in the ability of Tfh cells to induce B cell differentiation into plasma cells. Thus, our study provides evidence of Tfh cells and IL-21 in the pathogenesis of AIH with elevated serum IgG4. IgG4-RD has a high recurrence rate after discontinuation of glucocorticoids [13]. Thus, we speculate that IL-21 blockade or inhibition of Tfh cell activation may provide new therapeutic targets for the treatment of liver damage with serum IgG4 elevation.

In summary, elevated IgG4 levels in patients with liver injury are commonly seen in almost all liver diseases, and the prominent liver damage is characterized by elevated GGT, indicating susceptibility to combined bile duct injury. Glucocorticoid therapy is effective in patients with liver damage caused by IgG4-RD. The concomitant diseases, changes in liver function, and complement in AIH patients with elevated serum IgG4 differed from those of AIH patients with normal serum IgG4, indicating different immune responses and different targets of action; Tfh cells and IL-21 may contribute to the pathogenesis of AIH with elevated serum IgG4. Studies on immune function in patients with liver injury and elevated serum IgG4 may be useful for diagnosis and treatment of the associated diseases. Further studies are required to fully elucidate the mechanisms of Tfh cell activation, IL-21 production, and liver injury caused by the target cells of the immune response. In addition, it remains to be determined whether the pathogenesis of liver injury with elevated serum IgG4 is the same as that of IgG4-RD. The information presented here should prove useful and effective for improving the diagnosis and treatment of abnormal liver function with elevated serum IgG4 levels.

## Figures and Tables

**Table 1:** Abnormal liver function indices of 136 patients with elevated serum IgG4.

	Number of patients with normal liver function indices	Number of patients with abnormal liver function indices			
		1- to 5-fold ULN (%)	5- to 10-fold ULN (%)	>10-fold ULN (%)	Total (%)
ALT (0–40 U/L)	61	49 (65.3)	15 (20.0)	11 (14.7)	75 (55.1)
AST (0–40 U/L)	67	52 (75.4)	8 (11.6)	9 (13.0)	69 (50.7)
ALKP (40–150 U/L)	74	60 (96.8)	2 (3.2)	0 (0)	62 (45.6)
GGT (7–49 U/L)	18	76 (64.4)	18 (15.3)	24 (20.3)	118 (86.8)
TBil (3.4–20 *μ*mol/L)	67	53 (76.8)	11 (15.9)	5 (7.2)	69 (50.7)
DBil (0.1–6.8 *μ*mol/L)	54	56 (68.3)	9(11.0)	17 (20.7)	82 (60.3)

**Table 2:** Comparison of AIH patients with elevated serum IgG4 with those with normal IgG4 levels.

Characteristics	AIH with elevated serum IgG4	AIH with normal serum IgG4	*P* value
	19	20	—
Male	10 (52.6%)	4 (20.0%)	0.048
Age (≤65 yr)	14 (73.7%)	15 (75.0%)	1.000
IgE elevated	8 (42.1%)	2 (10.0%)	0.031
C3 decreased	12 (63.2%)	9 (45.0%)	0.341
C4 decreased	14 (73.7%)	3 (15.0%)	<0.001
ALT ≥ 10 times ULN	8 (42.1%)	2 (10.0%)	0.031
AST ≥ 10 times ULN	8 (42.1%)	2 (10.0%)	0.031
Allergic disease	8 (42.1%)	2 (10.0%)	0.031
Constipation	8 (42.1%)	2 (10.0%)	0.031

## Data Availability

The data used to support the findings of this study are available from the corresponding author upon request.
